# Supplementary material for: Sex-Dependent Effects of Piromelatine Treatment on Sleep-Wake Cycle and Sleep Structure of Prenatally Stressed Rats
Source: Int J Mol Sci. 2022 Sep 8;23(18):10349. doi: 10.3390/ijms231810349 (PMC9499655; doi:10.3390/ijms231810349)
Supplement: Supplementary file 1 [file ijms-23-10349-s001.zip › Suppl. Figures 1-3.pdf]

Supplementary Figures

Figure S1

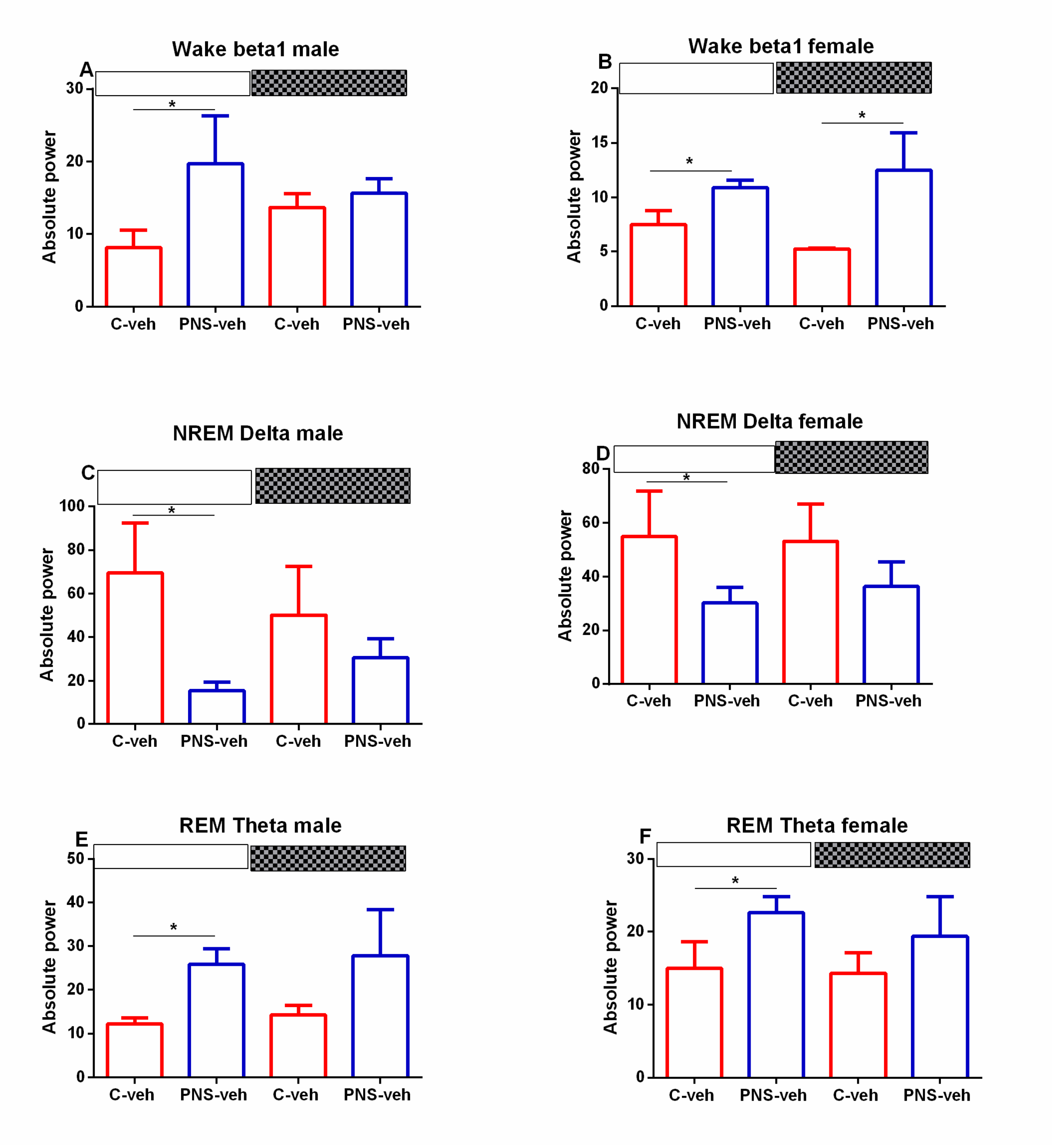

Figure S2

A)

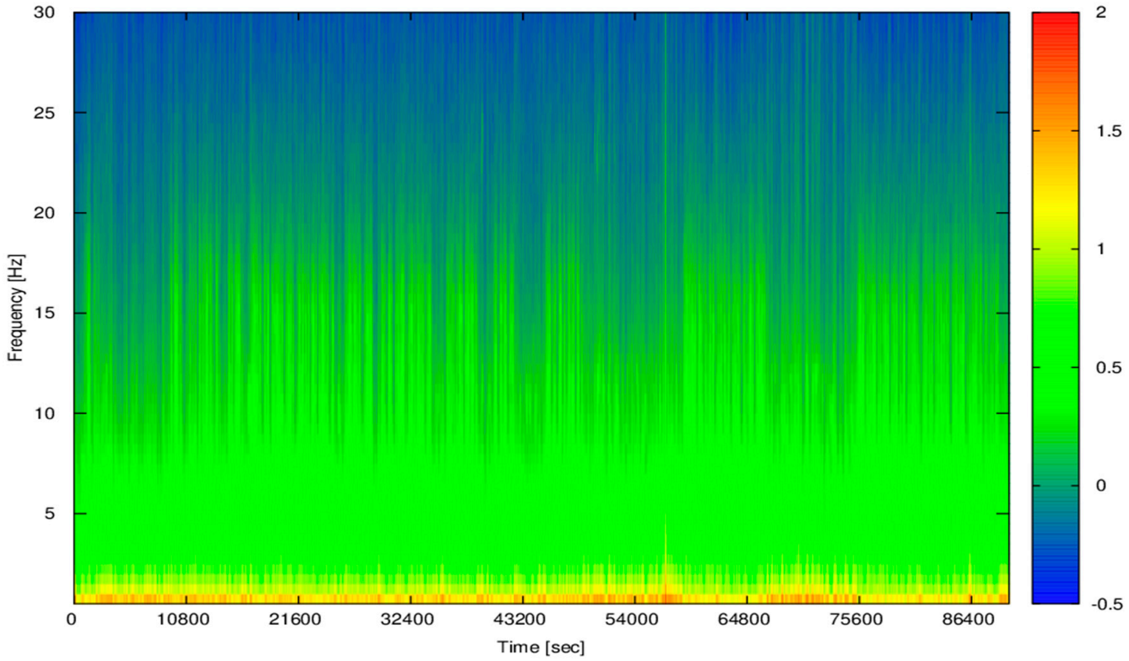

B)

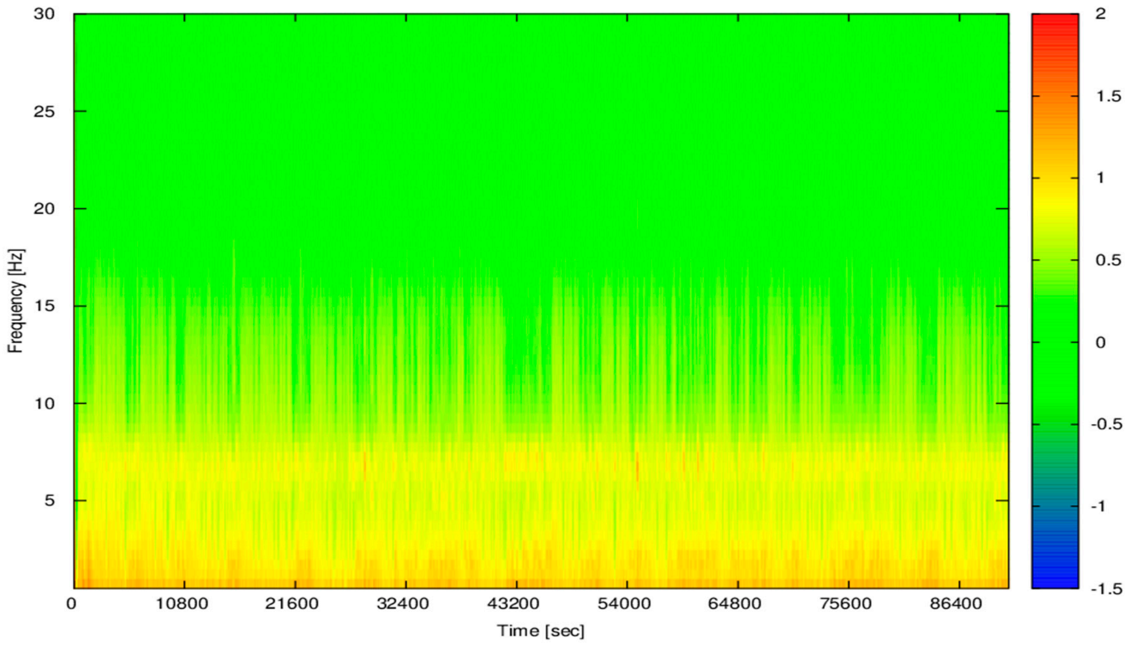

C)

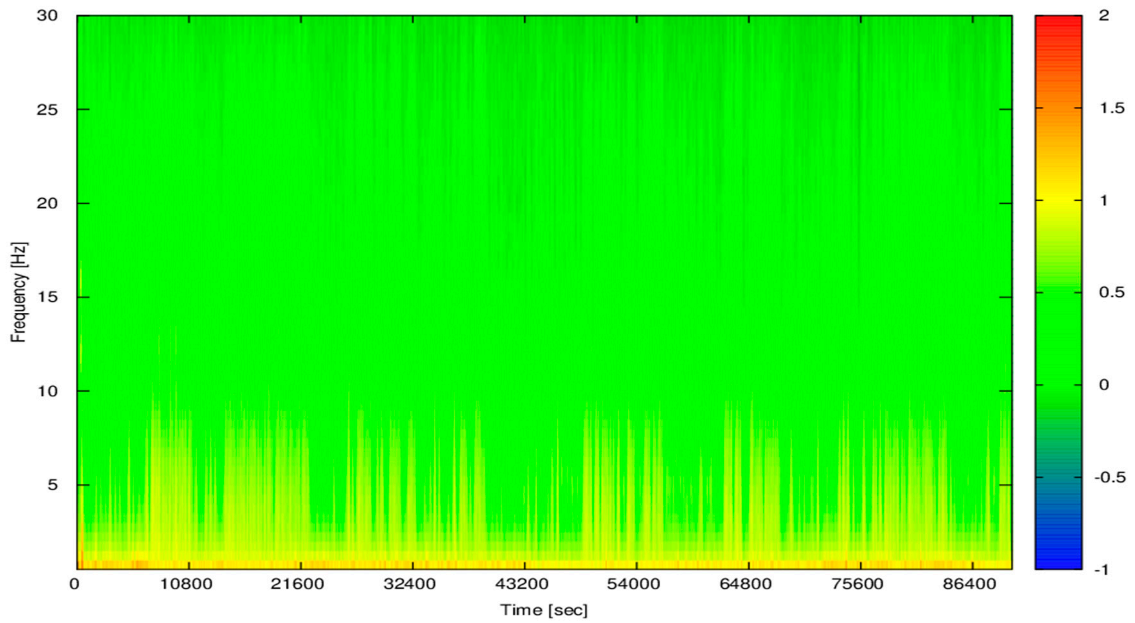
Frequency [Hz]
30
25
20
15
10
5
0
10800
21600
32400
43200
54000
64800
75600
86400
Time [sec]
2
1.5
1
0.5
0
-0.5
-1

Figure S3

A)

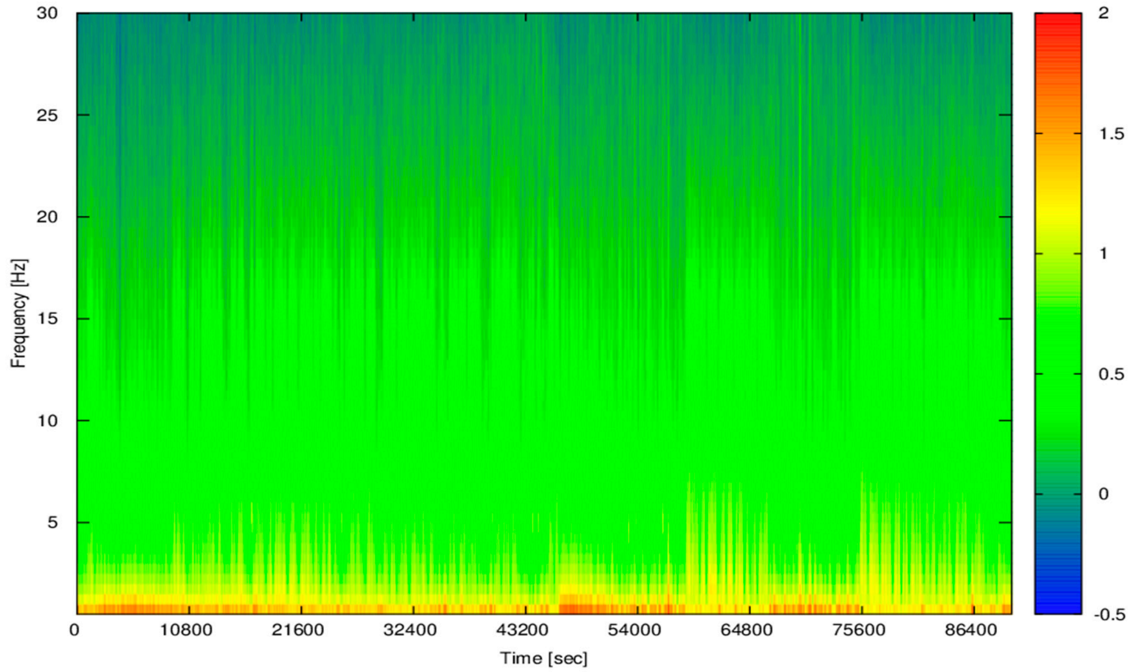
Frequency [Hz]
30
25
20
15
10
5
0
10800
21600
32400
43200
54000
64800
75600
86400
Time [sec]
2
1.5
1
0.5
0
-0.5

B)

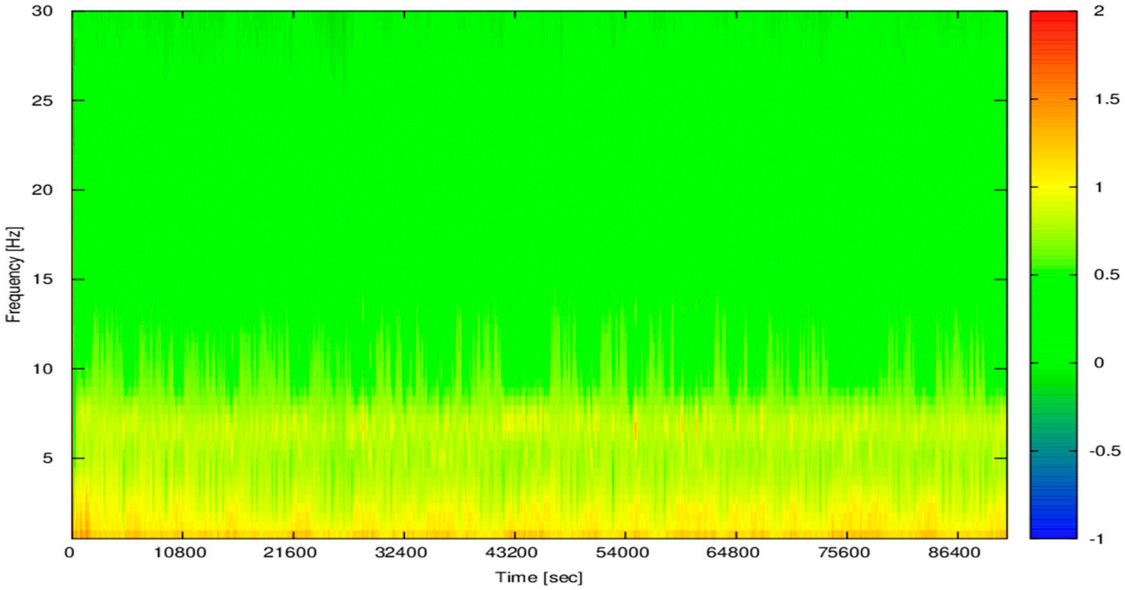
30
25
20
15
10
5
Frequency [Hz]
0
10800
21600
32400
43200
54000
64800
75600
86400
Time [sec]
2
1.5
1
0.5
0
-0.5
-1

C)

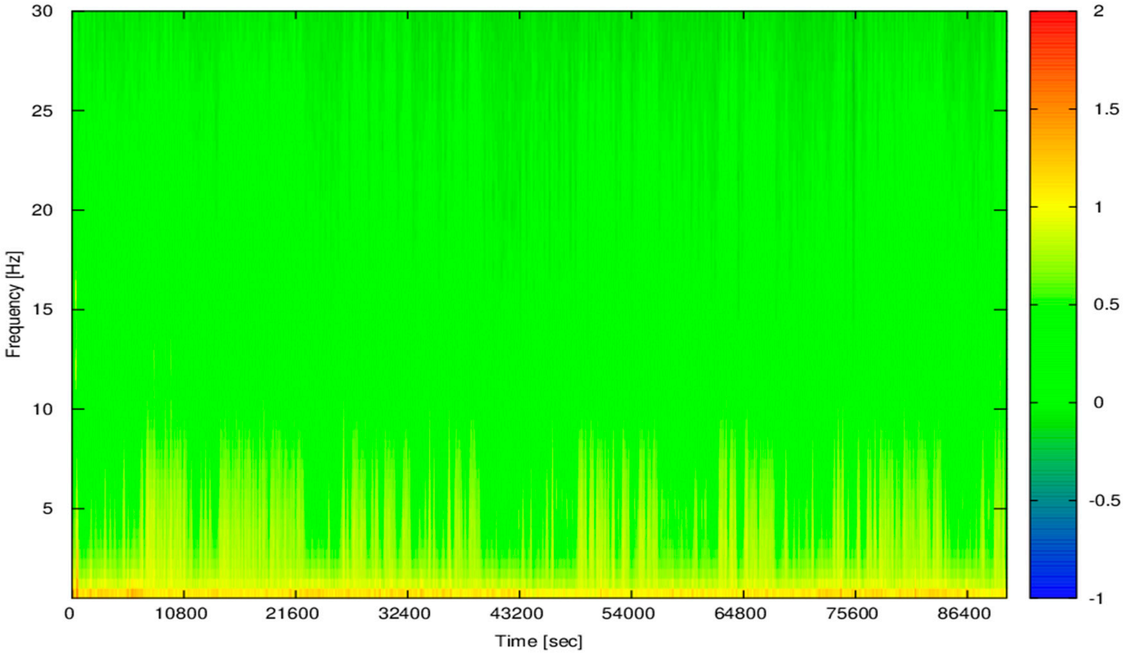
30
25
20
15
10
5
Frequency [Hz]
0
10800
21600
32400
43200
54000
64800
75600
86400
Time [sec]
2
1.5
1
0.5
0
-0.5
-1

Text to figures

Supp. S1 The effect of prenatal stress on spectral power of beta-1, delta, and theta EEG bands analyzed during the 24-h period in male and female rats, respectively. Ordinate: percent of a total power of beta-1 bands (14.5–18.6Hz), delta bands (0.5– 4 Hz), and theta bands (4–8 Hz). Data are presented as mean ± SD. *$p < 0.05$ vs. LD-group

Suppl. S2 Representative multi-taper spectrogram from the left+right FC over a 24-h recording for male A) C-veh group, B) PNS-veh, C) PNS-Pir group, respectively, during the light phase of the light-dark cycle. Different colors show different power values (see left legend bar). Ordinate: frequency; Abscissa: time.

Suppl. S3 Representative multi-taper spectrogram from the left+right FC over a 24-h recording for female A) C-veh group, B) C-Pir, C) PNS-veh, D) PNS-Pir group, respectively. Different colors show different power values (see left legend bar). Ordinate: frequency; Abscissa: time.
