# Supplementary material for: Sex-Dependent Effects of Piromelatine Treatment on Sleep-Wake Cycle and Sleep Structure of Prenatally Stressed Rats
Source: Int J Mol Sci. 2022 Sep 8;23(18):10349. doi: 10.3390/ijms231810349 (PMC9499655; doi:10.3390/ijms231810349)
Supplement: Supplementary file 1 [file ijms-23-10349-s001.zip › Suppl. Tables 1-8.pdf]

Supplementary

Table S1 Cosinor analysis of 24-h home-cage motor activity of male rats with piromelatine treatment and postnatal stress. The differences were analysed by multiple t-tests corrected using the Holm-Sídak method.

Group	Mesor	Amplitude	Acrophase
C-veh	116.46	83.12	16.83
C-Pir	101.42	70.50	17.91
PNS-veh	201.19	107.70	18.25
PNS-Pir	153.43	75.79	16.76

Table S2 Cosinor analysis of 24-h home-cage motor activity of female rats with piromelatine treatment and postnatal stress. The differences were analysed by multiple t-tests corrected using the Holm-Sídak method.

Group	Mesor	Amplitude	Acrophase
C-veh	187.93	78.80	16.23
C-Pir	166.40	99.14	16.60
PNS-veh	255.75	184.70	17.83
PNS-Pir	197.41	77.45	15.86

Table S3 Cosinor analysis of 24-h Wake activity detected by EEG recording of male rats with piromelatine treatment and postnatal stress. The differences were analysed by multiple t-tests corrected using the Holm-Sídak method.

Group	Mesor	Amplitude	Acrophase
C-veh	1577.73	539.36	15.14
C-Pir	1644.71	786.17	16.79
C-Pir-Luz	1886.34	321.09	15.73
PNS-veh	2289.38	257.99	8.72
PNS-Pir	1723.69	683.08	16.34
PNS-Pir-Luz	2054.83	206.23	8.82

Table S4 Cosinor analysis of 24-h Wake activity detected by EEG recording of female rats with piromelatine treatment and postnatal stress. The differences were analysed by multiple t-tests corrected using the Holm-Sídak method.

Group	Mesor	Amplitude	Acrophase
C-veh	1474.70	464.39	16.67
C-Pir	1401.75	404.74	13.59
C-Pir-Luz	1774.65	293.03	15.80
PNS-veh	2341.39	280.33	14.52
PNS-Pir	1244.24	340.75	16.70
PNS-Pir-Luz	1996.89	441.20	12.68

Table S5 Cosinor analysis of 24-h NREM activity detected by EEG recording of male rats with piromelatine treatment and postnatal stress. The differences were analysed by multiple t-tests corrected using the Holm-Sídak method.

Group	Mesor	Amplitude	Acrophase

C-veh	1816.05	905.72	4.16
C-Pir	1952.51	804.74	5.38
C-Pir-Luz	1572.33	357.22	11.28
PNS-veh	1483.82	411.21	4.03
PNS-Pir	2160.16	426.27	5.44
PNS-Pir-Luz	1259.14	141.50	0.17

Table S6 Cosinor analysis of 24-h NREM activity detected by EEG recording of female rats with piromelatine treatment and postnatal stress. The differences were analysed by multiple t-tests corrected using the Holm-Sídak method.

Group	Mesor	Amplitude	Acrophase
C-veh	2090.03	685.28	5.60
C-Pir	2034.23	875.88	4.43
C-Pir-Luz	1572.32	357.26	11.29
PNS-veh	1383.11	211.35	3.69
PNS-Pir	2227.53	397.44	4.12
PNS-Pir-Luz	203.68	192.02	10.22

Table S7 Cosinor analysis of 24-h REM activity detected by EEG recording of male rats with piromelatine treatment and postnatal stress. The differences were analysed by multiple t-tests corrected using the Holm-Sídak method.

Group	Mesor	Amplitude	Acrophase
C-veh	1474.70	464.39	16.67
C-Pir	335.74	208.23	7.62
C-Pir-Luz	106.91	10.49	3.38
PNS-veh	278.02	206.72	6.84
PNS-Pir	155,05	118.42	8.35
PNS-Pir-Luz	155.04	118.42	8.34

Table S8 Cosinor analysis of 24-h REM activity detected by EEG recording of female rats with piromelatine treatment and postnatal stress. The differences were analysed by multiple t-tests corrected using the Holm-Sídak method.

Group	Mesor	Amplitude	Acrophase
C-veh	418.09	281.93	5.26
C-Pir	335.74	208.03	7.68
C-Pir-Luz	414.26	131.23	13.70
PNS-veh	1825.70	1026.90	0.45
PNS-Pir	263.35	30.14	17.04
PNS-Pir-Luz	650.09	19.17	19.43
